# Report of the Kentucky State Dental Association

**Published:** 1880-08

**Authors:** 


### REPORT OF THE DISCUSSIONS BEFORE THE KY. STATE DENTAL ASSOCIATION, JUNE 2ND AND 3RD, 1880.

*Subject for Discussion*—ANATOMY. *The Committee had no paper on the subject.*

*Dr. Rawls.*—Mr. President, this subject is one of very great importance, and it has been very much neglected. It has not been neglected by Anatomists, but by the profession. We have not seemed to feel how important it is to understand, not only the Anatomy of the head, but minute Anatomy, that we may understand the effect of disease upon all the parts. For the want of this knowledge, we are frequently at a loss to know what to do—one of the most difficult things I had to contend with when I commenced practice, was to understand Pathology.

We do not dip deeply enough into minute anatomy, histology, &c., and without this we can not understand the diseased condition of the parts—it is the foundation of a knowledge of diseased conditions—and I would be glad to see this subject thoroughly presented and discussed in such a way, that the profession might realize its great importance and value.

*Dr. Taft.*—Mr. President, I have on this subject only a suggestion to make. When we speak of Anatomy, we are too apt to fix the attention more upon the grosser structures of the parts, but we should give more attention to minute anatomy, microscopic anatomy; this subject we should discuss and present so as to stimulate others in this work; there are many ways we can study Anatomy without the human body, and quite as well too. Frogs, Fowls, and most of the lower animals may be utilized in this way. Get a microscope and study these things. Every dentist, no matter how busy, can find some time to study in this way, I am sure. Avail yourself of the microscope, and under the direction of Beel, Frey and such men, you can study the growth and development of the parts, circulation, &c. Every dentist should have a microscope. Microscopes and all the necessary appliances have become so cheap now, that every one can procure them. Take the eye of the ox for instance. why we can spend days in its study. Take the head of the calf or the sheep, the adult, the young, the fœtal; examine the teeth and all the parts. It is not for the want of time or opportunity to study these things, it is simply because we do not avail ourselves of the opportunity we have. The society is not the place to study such things, but simply to bring them up and talk them over, and try and stimulate each other to study the subject.

Subject passed.

PATHOLOGY.—Committee Peabody and Edwards.

*Dr. Peabody.*—Mr. President, for the first time in six years, I appear before this association without a paper, but circumstances have been such, that it has been impossible for me to prepare one. I made several attempts, but it seemed impossible for me to collect my thoughts sufficiently; even last week I tried to jot down some few things to present before you, but I gave it up.

I feel somewhat ashamed that I have not a paper, but I could not help it.

Not having a paper, I suppose I should say something on the subject, and yet I hardly know what to say, the field is so broad, it is very difficult to pick on just what would be most interesting. I have had nothing in my own practice that I could make a paper on, but I have one or two cases I would like to mention, not that they are strictly connected with the subject, but simply to relate them that others may speak of them. The first case was brought to me by one of our physicians, of Louisville, last fall. He was unable to give me a complete history of the case. It was this: A gentleman was brought into my office, face very much swollen, he seemed to be in great pain. He was a man of very large physique. He told me that one day in biting a radish, his teeth slipped through very suddenly. (The radish was soft, pithy), struck the gum and wounded it, at the time it gave him no inconvenience, and he thought little of it. After several days it gave him great pain, his physician gave him some simple wash at first, but the inflammation continued to extend, and was very great. The physician treated for Erysipelas, but could give him no relief. He had lanced the jaw at the Symphysis, at the ramus of the jaw and even down at the shoulder—the pus flowed freely at each opening. I found the teeth all very loose, and pus oozing out from around all the teeth. I removed the first bicuspid, not a drop of blood followed. I found the root covered with pus and denuded of periosteum. I introduced the probe into the socket, could feel necrosed bone. I suggested the removal of the second bicuspid, but the physician objected; after several days he came back, when I removed it, and found the root perfectly smooth. I introduced the probe, but failed to get it to pass through. I suggested an operation. The physician told me he had presented the case to a surgeon, and he thought it would be better to defer it, as the weather was very warm, and it would be better to wait for cooler weather. I think myself this was very well. I then suggested the use of Sulphuric Acid. I made one application of Arom. Sul. Acid, and in a few days the pus ceased, and he improved.

Sometime in January he came to see me again, and at my suggestion, he permitted me to remove the first inferior molar; the physician thought I had done wrong, but I did not think I had. I introduced the probe at the opening made at the ramus of the jaw, and felt necrosed bone. The physician told me he had removed large pieces of necrosed bone. Had the physician given me the history of the case as he promised, I would have written an account, and had it better presented before you, but the whole trouble commenced with the biting of the radish.

The second case, was that of an Irishman, who fell from the fourth story of a warehouse, as has been said in connection with two other barrels of whisky. I don't know whether he was a drinking man or not, don't think he was though, as Mr. Buchanan is very particular, and I don't think he would have kept him if he had been a very hard drinker. The man was on the elevator with two barrels of whisky, when the rope broke, and they went to the bottom. He was hurt very badly, and considerably bruised about the face and head, his chin was cut and the teeth driven in so they hooked under the upper ones very much, I brought the parts together as well as I could, the teeth moved with the bone, and when I got the parts together, I found I had brought the teeth out considerably, I ligatured the teeth together but could not bring them out, I took a cast of the mouth and made a plate, and brought the teeth to normal occlusion, these three teeth struck inside, but the lower were loose. He came back in five or six days, there was no union, no effort at union. The interesting feature of the case, is, what was the condition of the bone? there was no necrosis and how nature tolerated such a condition without necrosis or union it is difficult for me to understand, it could not have been the condition of health. The man has gone to work again.

The third case is that of a lady, for whom I put in two fillings in the approximal surfaces of the first inferior molar and second bicuspid, in about six weeks she returned, complaining that they were hurting, I examined them and found swelling hard, indurated; treated but gave no relief; removed filling; first molar very sensitive when filled; cut down; struck the nerve; passed into the pulp; bled freely; treated some time; the enlargement went down; I removed the filling from the bicuspid; found nerve dead; enlarged nerve canal and found foramen closed, I drilled through and the swelling went down, but I have not been able to reduce the enlargement, it is not an outgrowth, it appears as though the outer plate of bone had been forced out, seems hardly possible that the inferior jaw should be forced out in that way, bone of such density; there is enlargement there yet, though only about one-half what it was, these are cases I present for consideration and to get other's views of them.

*Dr. Rawls.*—Mr. President, I am not a member of the committee, but I have been very much interested in the cases as related by Dr. Peabody. It is just such cases as these we are all interested in, and the profession should be doubly interested because we know so little, as to how to handle them, sometimes with apparently simple cases, we are at a loss to know how to treat them. My mind was struck with the obstinacy of two of the cases given by Dr. Peabody, in the first case, from the description, the vitality of the body was as good as we ordinarily see, and yet from a simple wound we have violent ulcers as would occur from lupus. In the other case, had we not been told the vitality was good, I would suppose the man to be a drinking man and from that cause rather favor disease.

What is the cause of this condition of things, here we have two cases diametrically opposite, in one case we have the jaw fractured, teeth apparently separated from living tissue, union takes place in one part.

It is this condition of things as shown in the case of Mr. Gardner, which has attracted so much attention. The case of Dr. Waters, here we have irritation, inflammation and even death of the parts. Arsenic we all know is a powerful irritant, but we know as the dentist generally uses it, it is not dangerous, what is this condition, that in one favors the breaking down of the tissues and death of the parts, I know some will say the body was in condition to receive the effects, but, what is the condition, is it known to the physician and the dentist, we should, it seems to me, be more thorough in removing all causes of irritation, we should favor the laws of gravitation in allowing pus to make its exit, when pus once accumulates about the bone it is sometimes a hard matter to get rid of it, the absorbents are not numerous; void the pus as much as possible, get rid of it by the laws of gravitation.

The third case occurred just as the first case, except in this, the abscess being surrounded by dense bone, the pus caused absorption of the outer plate until it became very thin and caused expansion and produced the protuberance.

*Dr. Peabody.*—I introduced a syringe in the nerve canal and exhausted every particle of moisture, there was no pus or moisture there.

*Dr. Rawls.*—That don't indicate that there was not pus there.

*Dr. Peabody.*—There was no soreness—no looseness.

*Dr. Rawls.*—We often have cases where we are not able to know the first condition, I think the third case was at one time cured. I would like to hear a full discussion of the first two cases.

*Dr. Peabody.*—I would like to ask in this case, what would be the result of introducing a drill in what appeared a cavity, what would be the result of such treatment?

*Dr. Rawls.*—I can't tell, that would depend on the condition of things, you might find pus, you might not, you might make an opening and not find anything, it is a very singular case.

*Dr. Van Antwerp.*—It being situated in unyeilding parts, it may have been, there was'nt any chance for the pus to pass out.

*Dr. Peabody.*—I would like to hear from Dr. Van Antwerp.

*Dr. Van Antwerp.*—Mr. President, the subject of pathology is one of great interest, I don't know that I can throw any light on the subject, it is one in which I am about as blind as any one. Speaking of these cases, reminds me of a case I treated one time for eighteen months, I attributed the trouble to lack of vitality, however, the patient recovered but lost the jaw, it was attributed to my lack of skill, but I have the satisfaction of having the endorsement of Dr. Drake, one of the best physicians in our section of country, and Dr. Dawson of Cincinnati.

The case was, a little girl about eight or nine years of age, I extracted the second temporary molar, I could have removed it with my fingers but thought I had better not try it and took the forceps, I started across the room for some water, had not got more than half way across when the child cried out in great pain, she complained of no pain in extracting and I could not find any reason for the pain then. She went home, and the next day Dr. Drake called at my office and told me I had better go down and see her, I went down, found her suffering very much, the jaw swollen, I examined the case thoroughly, felt the new tooth, I told the mother it was an abscess, she was very anemic, it went on, the abscess broke. I was not called to the case any more, Dr. Drake feared cancer. Dr. Dawson operated on her and removed the necrosed bone in which were two teeth ossified and only partly developed, under the influence of tonics, cod liver oil, &c., the patient recovered. I attributed the whole dfficulty to lack of vitality, I watched the case and shall continue to watch it with great interest.

*Dr. Taft.*—The cases presented by Dr. Peabody are full of interest, especially the first two. The excessive suppuration at times is rather peculiar. Every surgeon is familiar with the fact that the head and neck, from their construction, are rather favorable to this. The patients in such affections are usually nervous. It is not so much the cause of this affection with which we are just now interested, as to assertain what should be done to meet the emergency. Many times too much importance is given to the cause. In many cases physicians are called to see cases in which they can't decide as to the cause, but may easily decide what should be done. In this case, undoubtedly, there was excessive suppuration--if there is necrosed bone; take that away. This can be done in various ways. Many times there will be great breaking down of both soft and bony tissues; the important question to be determined is how to remove it. This is of a great deal more importance now to the man who treats the case, than the cause. If separation of the bone is indicated by the appearance of the parts—if it moves—the first thing is to remove it; take it away, no matter how much you have to cut the soft parts, don't wait for it to work its way out. Many times there is not energy enough in the parts to throw off dead bone; stimulate the parts to healthy action, when this is accomplished reparation will take place. Many times in these parts you will have pockets formed; sometimes you will have a pool of pus, which will keep up irritation, open all such and open freely, then use local treatment of a stimulating kind, use antiseptics. I apprehend the case treated in this way would soon improve. The whisky case is rather singular. How were the soft parts? How were they around the teeth?

*Dr Peabody.*—Line of gum good—perfect union.

*Dr. Taft.*—That portion of the detached part must have retained some considerable vital connection, that is the reason why there was not more inflammation of the gum. Living tissue does not remain in contact with dead bone without irritation. I I apprehend he was not a drinking man—shouldn't wonder if he was a teetotaler. I think you will find by and by that irritation will cause wasting away. If the bone could be fixed in proper place, it would probably unite well, it would be very difficult without it, being subject to oscillation, and I think sooner or later wasting away will take place.

In the third case I would puncture the enlargement, and then inject with iodine.

*Dr. Peabody.*—In the second case, there is no question in my mind, if we could cause union of the bone to take place all would be right; there is one thing certain, the condition of things can't remain as they are; you cannot retain dead bone with living tissue. I am satisfied the bone is not dead, if it is, exfoliation will take place. In the first case, it makes no difference what the cause was. There may have been some blood trouble; it may have been in just such condition, that all it wanted was the wound, to start the trouble. I knew a gentlemen once, who used to say that all he wanted was an abrasion of the skin to kill him. He afterwards did cut the skin and died from the effects; the trouble existed in the system. The operation I thought necessary in this case was refused by the surgeon. Nature will do a great deal, and there may be a deposit of osseous tissue thrown out there, or there may be discharge of pus and necrosis, and if it is not removed, he will probably lose the whole jaw.

*Dr. A. W. Smith*—I have a case of "osteo sarcoma." I hoped to be able to present the patient before the Association, but I have been unable to get him here as yet. He lives some miles up the railroad. I have written him but have no response. I could give you a partial history of the case, but would prefer you to see it. Dr. Sutphin, a physician from the lower portion of the State is present; I would like to hear from him.

*Dr. Sutphin.*—Mr. President: I did not expect to take any part in your discussions. I came here more to visit your country than I did to the Association; never having seen your country before.

I think it probable in the first case, there was some constitutional trouble. Many times physicians are called on to treat cases they cannot explain the cause, in any other way; there are many forms of this. We have different diathesis, we have strumous diathesis, inflammatory diathesis. I knew of a case of death once caused by an arrow—a toy arrow punctured the skin and death was the result; then again a simple scratch will cause death. These are generally from constitutional trouble. We cannot understand why one patient will tolerate so much and another will not.

*Dr. H. A. Smith.*—I was not present when the cases were presented, but I noticed in the discussion of case number two—I think it was—that one gentleman asserted that necrosed bone always sloughed off, that is not always so. We have some cases of fractured root, for instance, where the piece will be retained without vital connection, and remain so for years, without occasioning any trouble whatever. In this case, there might have been a false joint formed after the accident, the mobility of the parts being kept up, a false joint was formed.

I had a case in my own practice where the molars and bicuspids were forced inside. I saw the case immediately after the accident, brought the parts together, and in less than two weeks union took place. My point is this: that a false joint may be formed and such condition tolerated for years, and even for life.

*Dr. Taft.*—Some years ago, I remember in attempting to extract a second superior molar tooth; the bone forming the sockets of the second and third molars was fractured from the main body of the jaw; neither the tooth nor the broken bone were removed, but all permitted to remain. Little or no suppuration took place. I was surprised, of course, but all became attached and the teeth were found fixed in place. Had not union taken place it would all have come away. The condition was more favorable for union than in Dr. Peabody's case,more favorable for it to remain till union did take place.

*Dr. Canine.*—I have a case from my own practice, of which I intended to present a written history to the association, but came away from home and left the papers in my desk, it was a case of peculiar interest.

A gentleman who was wounded by the explosion of the boiler of a saw mill, he was one hundred and twenty miles from Louisville and laid there two months, when he was brought to Dr. D. W. Yandell who brought him to me. It was a compound fracture of the lower maxillary, it was fractured in three places, first on the left side, between the first and second molars, on the right side there was an oblique fracture extending from the first molar backward and downward towards the third molar, the first molar having been extracted previous to the accident, the other fracture was at the left lateral incisor which was knocked out and lost, the bone on the left side was knocked outward so that it stood almost on a straight line with the cheek, that portion of the jaw on the right side was knocked in and down, and at the fracture at the incisor the bone had slipped behind the left portion, this point remained loose, partial union had taken place at the points of fracture at the molars on both sides. The result was, when the jaw was closed so that the molars struck together, there was space enough between the upper and lower front teeth to pass the fingers between them. To bring the parts together, to correct the articulation of the teeth, it was neccessary to break the union at the molar teeth and raise up that portion of the bone that had been knocked down and in, this I did, which was not an easy matter, I assure you; after considerable difficulty I succeeded in bringing the parts together and restored the articulation of the teeth. I then made a platinum and gold band, fitting the inside of the front and bicuspid teeth with arms coming to the front at the space made by the loss of the lateral incisor, these I bent around the incisors and cuspid teeth which held it securely in position, I then made a copper cup to fit the outside of the jaw with brass bands to extend over the head, this cup I lined or cushioned with "White's modeling material for impressions," thus securing perfect adaptation to the parts with no danger of rubbing or wounding the skin of the face, I then placed the appliance and tied the bands with strong tape, thus holding all the parts firmly in position. The gentleman came to see me first on the 17th of February, I saw him on the 27th of April, he was then doing well, he had not suffered any more inconvenience than you would naturally expect under the circumstances.

[TO BE CONTINUED.]

